# Isolated Roux loop pancreaticojejunostomy versus conventional pancreaticojejunostomy after pancreaticoduodenectomy: A case-control study

**DOI:** 10.1016/j.ijscr.2018.10.037

**Published:** 2018-10-31

**Authors:** Amine Chhaidar, Mohamed Ben Mabrouk, Ali ben Ali

**Affiliations:** Department of Surgery, Sahloul Hospital, Sousse, Tunisia

**Keywords:** Pancreaticoduodenectomy, Pancreaticojejunostomy, Isolated Roux loop, Pancreatic fistula

## Abstract

**Objectives:**

The aim of this study was to compare the postoperative outcomes of isolated Roux loop pancreaticojejunostomy (IPJ) and conventional pancreaticojejunostomy (CPJ) after pancreaticoduodenectomy (PD).

**Methods:**

Data of patients who underwent IPJ were compared with those of a pair-matched equal number of patients undergoing CPJ. The matching was performed according to age, gender, nature of the lesion indicating PD and the texture of the pancreas. The primary outcome was the rate of postoperative pancreatic fistula (POPF). Secondary outcomes included operative time, day to resumption of oral feeding, postoperative morbidity and mortality.

**Results:**

Seventy patients treated by PD (35 patients in each group) were included in the study. The two groups were comparable with regards to the pre-operative and intra-operative parameters. Postoperative pancreatic fistula developed in 10 out of 35 patients in the CPJ group and 3 out of 35 patients in the IPJ group (*p* =  0.031). Nine CPJ patients and one IPJ patient had POPF of type B or C (*p* =  0.006). Re-laparotomy was significantly more frequent in the CPJ group (11.1% versus 34.6%; *p* = 0.04). Time to resumption of oral feeding was shorter in the IPJ group (*p* =  0.001).

**Conclusions:**

The use of IPJ is associated with decrease in the rate of postoperative PF in patients undergoing PD. In addition, patients with IPJ reconstruction have lesser need for re-laparotomy and early resumption of oral feeding.

## Highlights

• Pancreaticojejunostomy is commonly used in the reconstruction after pancreaticoduodenectomy, but the incidence of POPF remains high.
• There are a number of theoretical advantages to the isolated Roux loop pancreaticojejunostomy reconstruction, mainly related to the physical separation of bile acids and the pancreaticojejunostomy.
• The use of an isolated Roux loop pancreaticojejunostomy seems to be associated with decrease in the rate of postoperative PF in patients undergoing PD.

## 1 Introduction

Pancreaticoduodenectomy (PD) is regarded as one of the most challenging and complex intra-abdominal operations. Although postoperative mortality has decreased to less than 5% in major centers, the rate of postoperative complications remains high and is largely due to breakdown of the pancreatic anastomosis [[1], [2], [3], [4]].

Postoperative pancreatic fistula (POPF), ranging from 5% to 25% in most series, is considered to be one of the most serious complications after PD [5]. Severe POPF can even cause intra-abdominal abscesses, sepsis and life-threatening hemorrhage. One of the contributors to the significant variations in the observed incidence of POPF between the medical centers may be related to the technique of reconstruction. There is ongoing interest in the development of techniques to reduce the rate of pancreatic anastomotic leakage and its associated morbidity. Numerous methods to deal with the pancreatic remnant have been proposed, including use of fibrin glue to occlude the main pancreatic duct [6], suture ligation of the pancreatic duct [7], pancreatic duct stenting [8], modification of the jejunal anastomosis (end-to-end versus end-to-side, invagination versus duct-to-mucosa) [9,10], pancreaticogastrostomy [11] and use of a Roux loop to drain the pancreas [12]. Currently, the optimal surgical technique to reduce POPF is unknown [13].

Pancreaticojejunostomy is commonly used in the reconstruction after PD, but the incidence of POPF remains high [14]. An isolated Roux loop pancreaticojejunostomy (IPJ) for the drainage of the pancreatic stump was first proposed by Machado et al. in 1976 [15]. In a conventional pancreaticojejunostomy (CPJ), both hepaticojejunostomy (HJ) and gastrojejunostomy (GJ) is performed in the same continuous limb of small bowel. Whereas in IPJ, pancreaticojejunostomy is fashioned on a separate (or isolated) limb, joining the other limb by jejunojejunostomy. Proponents of this technique believe that the diversion of bile away from the pancreaticojejunostomy site minimizes the pancreatic enzyme activation and thus reduces the risk of POPF [[15], [16], [17]]. Although this surgical procedure has some theoretical advantages, only few studies have reported the use of IPJ. Some non-comparative studies have reported lower rates of POPF after IPJ [16,17]. Recent comparative studies, however, found no difference between IPJ and CPJ [12,[18], [19], [20]].

Because of the controversies in the published literature on the potential superiority of IPJ over CPJ after PD, we analyzed the impact of IPJ and CPJ on the occurrence of postoperative PF using a case-matched analysis of our patients to balance the selection bias.

## 2 Methods

### 2.1 Study design and ethical standard

This study was a single-center, retrospective observational study with prospectively collected data. This study was in accordance with the ethical standards of the institutional and national research committee and with the 1964 Helsinki declaration and its later amendments. The research registry number in accordance with the declaration of Helsinki is 3491. This study was approved by the relevant Ethics Committee and informed consent was obtained from all patients. The research work has been reported in line with the PROCESS criteria [21].

### 2.2 Patients

This study is a retrospective analysis of patients undergoing PD by a single surgical team between 1 st January 2005 and 31 December 2015 at Department of surgery, Sahloul Hospital, Sousse, Tunisia. For the purpose of analysis, all patients in whom an IPJ was performed were grouped together (Group 1). These patients were compared with a pair-matched equal number of patients undergoing CPJ with a single loop. The matching was performed according to age, gender, nature of the primary lesion indicating PD and the texture of the pancreas.

### 2.3 Operative details

All patients underwent classical Kausch–Whipple PD. A small jejunotomy was made corresponding to the pancreatic duct diameter. Pancreaticojejunostomy was performed with 4/0 interrupted silk sutures between the capsule of pancreas and the seromuscular layer of jejunum without pancreatic stenting.

In group 1 (IPJ), a 40-cm isolated loop of jejunum was passed through the mesocolon for PJanastomosis. The remaining reconstruction was completed by end-to-side bilioenteric anastomosis followed by GJ from the distal loop of jejunum and the jejunojejunostomy was constructed after GJ.

In group 2 (CPJ), the reconstruction was performed using a single, retro colic jejunal loop. Pancreaticojejunostomy was performed first, followed by HJ and posterior GJ at a distance of 15–20 cm each. We routinely measured drain fluid amylase on postoperative day 1, 3 and 7.

### 2.4 Data collection and study definitions

Detailed information about these patients was maintained in a prospective database including demographics, American Society of Anesthesiology (ASA) scores and the presence of comorbidities (hypertension, or diabetes mellitus).Preoperative imaging included abdominal ultrasound, contrast-enhanced computed tomography (CT) or magnetic resonance imaging of the abdomen and endoscopic retrograde cholangiopancreatography. Intraoperative features, such as tumor size, pancreatic duct diameter, consistency of pancreas, type of pancreaticojejunal (PJ) anastomosis, duration of surgery and blood loss were carefully documented.

Postoperative pancreatic fistula was defined according to the International Study Group on Pancreatic Fistula (ISGPF) definition: drainage of any measurable volume of fluid with fluid amylase more than or equal to three times the upper limit of normal serum value on postoperative day 3 or later [5]. Pancreatic fistulae were graded according to ISGPF criteria into Grades A, B and C according to their clinical course. The International Study Group on Pancreatic Fistula (ISGPF) definition for PF [5] was used retrospectively in patients operated before July 2005 on the basis of the information recorded in our prospective database. All cases of postoperative PF were identified as representative of either pure or complex fistulae depending on the nature of the draining fluid. The presence of bile or enteric contents suggested a complex fistula, whereas the presence of clear, water-like fluid suggested a pure PF. In case of suspicion of intra-abdominal sepsis, CT scans were liberally performed.

Delayed gastric emptying (DGE) was defined as the need for nasogastric intubation for >10 postoperative days, inability to resume enteral feed at 14 days after surgery, need of prokinetics for >10 days or re-insertion of the nasogastric tube [22].

Length of hospital stay was defined as the period from the first postoperative day until discharge from hospital. Death within the same hospital admission or within 30 days of surgery was considered as operative mortality.

### 2.5 Outcomes

The primary outcome was the rate of POPF. Secondary outcomes included total operative time, length of postoperative stay, postoperative complications (including delayed gastric emptying, biliary leakage, hemorrhage and pulmonary complications), endocrine and exocrine functions, need for re-exploration and day to resumption of oral feeding.

### 2.6 Statistical analysis

Statistical package for Social Sciences (SPSS version 20.0; IBM, USA) was used for data analysis. To test significance, Student’s *t*-test was used for continuous variables and chi-squared or Fischer’s exact test was used for categorical variables. A P-value of <0.05 was considered significant. The matching was performed according to age, gender, nature of the primary lesion indicating PD and the texture of the pancreas.

## 3 Results

During the study period, 130 PD were performed at our institution. Among 71 patients who underwent PD using IPJ, 35 cases were among the matching criteria, the 36 others were not matching this is why we did not include them into the study, and were compared with those of a pair matched equal number of patients undergoing CPJ. The characteristics of the two matched groups are presented in Table 1. Patient characteristics were well matched with no significant differences in age, sex ratio, presence of diabetes mellitus, presence of jaundice and preoperative biliary drainage. Intraoperative details are shown in Table 2. No significant differences were found in the pathology, pancreatic duct diameter and pancreatic consistency. Median operative time was not significantly longer in the CPJ group (5.5 h [range, 4–9.16 hours]) compared with the IPJ group (5.4 h [range, 4–8 hours]) (p = 0.84).

**Table 1:** Demographic data for patients submitted to isolated Roux loop pancreaticojejunostomy (IRPJ) or conventional pancreaticojejunostomy (CPJ).

	IPJ group (n = 35)	CPJ group (n = 35)	P value
Patient age, years, median (range)	61(44–74)	61 (44–74)	0,99
Sex, *n* (%)			
Female	23(65,7%)	23(65,7%)	1
Male	12(34,3%)	12(34,3%)	
Diabetes	7(20%)	9(25,7%)	0,56
ASA, n (%) I II III	16(47,1) 17(50%) 1(2,9%)	19(55,9%) 14(41,2%) 1(2,9%)	0,76
Symptoms, *n* (%) Jaundice	28(80%)	31(88,6%)	0,32
Abdominal pain	27(77,1%)	25(71,4)	0,58
Preoperative bilirubin, mmol/dl <250 >250 Preoperative biliary drainage, *n* (%)	21(77,8%) 14(22,2%) 2(5,7%)	25(71,4) 10(28,6) 3(8,6)	0,57 1

**Table 2:** Operative data for patients submitted to isolated Roux loop pancreaticojejunostomy (IPJ) or conventional pancreaticojejunostomy (CPJ).

	IPJ group (n = 35)	CPJ group (n = 35)	P value
Pathology, *n* (%) Pancreatic head mass Ampullary cancer	20(57,1%) 10(28,6%)	20(57,1%) 10(28,6%)	1
Lower CBD tumour	3(8,6%)	3(8,6%)	
Duodenaltumour	1(2,9%)	1(2,9%)	
Intraductal papillary neoplasms Pancreatic duct diameter, *n* (%) <3 mm >3 mm Pancreatic consistency, *n* (%) Firm Soft Total operative time, min, median (range)	1(2,9%) 11(31,4%) 24(68,6%) 17(48,6%) 18(51,4%) 325(240–480)	1(2,9%) 12(34,3%) 23(65,7%) 19(54,3%) 16(45,7%) 330(240–550)	0,79 0,63 0,84

Postoperative outcomes are shown in Table 3. Overall morbidity did not differ significantly between IPJ and CPJ groups (57.1% versus 52.1%; p = 0,7). PF occurred in three patients of IPJ group (8.6%) and in 10 (28.6%) patients of CPJ group (p = 0.031).The clinical PF (grade or C) was also significantly higher in the CPJ groups (25.7% versus 2.8%; p = 0.006).No statistical differences were found between the two groups with regard to other postoperative complications, including delayed gastric emptying, bile leakage and pulmonary complications. In the current study, oral feeding commenced 3 day earlier in the IPJ group than in the CPJ group. The length of postoperative hospital stay did not differ significantly between the two groups.

**Table 3:** Postoperative data for patients submitted to isolated Roux loop pancreaticojejunostomy (IRPJ) or conventional pancreaticojejunostomy (CPJ).

	IPJ group (n = 35)	CPJ group (n = 35)	P value	ODD ratio (95%CI)
Hospital stay, days, median (range) Time to oral feeding, days, mean (deviation) Patients with complications, *n* (%) Pancreatic leakage (POPF), *n* (%) Grade A, *n* Grade B, *n* Grade C, *n* Clinical fistula (grade B/C) *n* (%) Pancreatitis, *n* (%) Biliary leakage, *n* (%) Delayed gastric emptying, *n* (%) Internal haemorrhage, *n* (%) Pulmonary complications, *n* (%) Re-Laparotomy, *n* (%) Mortality, *n* (%)	10(6–49) 1,6 ± 0,9 18(52,9%) 3(8,6%) 2 0 1 1(2,8%) 1(2,9%) 2(5,71%) 0 1(2,9%) 2 3(11,1%) 3(8,5%)	13,5(10–27) 4,6 ± 1,9 20(57,1%) 10(28,6%) 1 3 6 9(25,7%) 2(5,7) 6(17,14%) 1(4,8%) 4(11,4%) 5 9(34,6%) 4(11,4%)	0,53 0,001 0,72 0,031 0,006 1 0,86 NS 0,3 0,1 0,041 1	0,119(0,03–0,43) 0,23(0,05–0,943) 0,085(0,01–0,71) 0,24(0,05–1)

## 4 Discussion

The postoperative morbidity following PD is approximately 30%–40% [2] and POPF remains the most important contributor [3]. A variety of techniques have been used and evaluated over the years, and IPJ reconstruction was designed specifically for the management of the pancreatic remnant after PD to reduce the incidence of POPF.

There are a number of theoretical advantages to IPJ reconstruction, mainly related to the physical separation of bile acids and the pancreaticojejunostomy. For example, pancreatic enzymes activated by bile could conceivably degrade suture material used in the pancreaticojejunostomy [23,24]. Also, bile reflux is well recognized as a factor associated with the induction of acute pancreatitis [25]. In fact, bile-diverting procedures can prevent activation of pancreatic secretions and therefore protect the pancreaticojejunal anastomosis. In addition, it can reduce mechanical stress on the pancreaticojejunostomy and reflux of biliary and gastric contents up the pancreaticojejunostomy.

On the other hand, the jejuno-jejunal anastomosis could cause edema to increase intraluminal pressure in the isolated limb and add the risk of occlusion with serious consequences for the pancreatic anastomosis, which is the most quoted theoretical disadvantage of IPJ [26,27].

Several case series studies have concluded that RYR is a safe procedure with a 0% leak rate [17,24,27,28]. However, all these studies lack appropriate controls, thus limiting the value of their conclusions. Hence, the aim of this study was to compare the outcomes of IPJ with those of CPJ after PD.

In many studies, IPJ did not avoid POPF but did decrease leak-related morbidity and mortality [12,17,19]. In a prospective randomized study of 216 patients, Ke et al. [12] found similar rates of POPF in patients undergoing conventional loop PJ and IPJ, but the ratio of Grade B POPF was much higher in the conventional PJ group than the IPJ group. In a meta-analysis of three randomized clinical trials and four controlled clinical trials including a total of 802 patients and comparing outcomes of CPJ and IPJ reconstruction after PD [29], there was no statistically significant difference in PF rates between the two reconstruction procedures across all studies (RR 0,91, 95% CI. 0,68 to 1,22; P = 0,54). Additionally, there was no significant difference between the two reconstructions for either overall morbidity and reoperations.

In the present case-matched analysis, there was statistically significant differences in PF(RR 0,23; 95%CI 0,05-0,94; p = 0,03) and clinically significant pancreatic fistula (grade B/C) (RR 0,085;95%CI 0,01-0,71;p = 0,006) between the two groups. Relaparotomy due to severe PF was significantly more often in the CPJ group. Analysis of postoperative mortality revealed no significant difference between the two groups.

Potential disadvantages of IPJ reconstruction are the need for an additional anastomosis and the risk of marginal ulcer development. The time required for the additional jejunojejunal anastomosis in the IPJ group was lessened with the use of a stapler, thus resulting in no significant differences in the operating time between the 2 groups.

In the current study, oral feeding could be commenced three days earlier in the IPJ group than in the CPJ group. Patients with POPF after IPJ resumed oral feeding without any increase in the amount of leak and demonstrated a principal advantage in the absence of delayed gastric emptying. However, some studies have found no significant difference in either of these factors [12,20].

The main limitations of our case-matched analysis are the degree of heterogeneity related to individual surgeons’ experience, which may have represented a source of bias and the small sample size. Besides, isolated Roux loop pancreaticojejunostomy was performed more recently comparing to conventional pancreaticojejunostomy.

## 5 Conclusions

The results of this study contribute to the growing evidence that isolated limb Roux-en- Y may be associated with a lower rate of postoperative pancreatic leak. Isolated Roux loop PJ also allowed for early oral feeding and facilitated the maintenance of oral feeding even if POPF developed. Nonetheless, further prospective randomized studies with larger sample sizes are required to confirm these results.

## Conflicts of interest

The authors declare that they have no conflict of interest.

## Funding

This study has not received any funding.

## Ethical approval

The study was approved by Ethics Committee of Hospital Sahloul.

## Consent

Written informed consent for the publication of this article was obtained from either the patient or their family. Copies of the written consents are available for review by the Editor-in-Chief of this journal.

## Authors’ contributions

Amine Chhaidar – data collection, Editing of manuscript.

Mohamed Ben Mabrouk – Editing of manuscript, literature review.

Ali Ben Ali – Editing of the manuscript.

## Registration of research studies

3491.

## Guarantor

Amine Chhaidar.

## Provenance and peer review

Not commissioned, externally peer reviewed.
